# High expression of RPL27A predicts poor prognosis in patients with hepatocellular carcinoma

**DOI:** 10.1186/s12957-023-03102-w

**Published:** 2023-07-21

**Authors:** Huiwu Xing, Xiangqi Jiang, Chenyu Yang, Bingqian Tan, Jiqiang Hu, Mingman Zhang

**Affiliations:** Department of Hepatobiliary Surgery, Children’s Hospital of Chongqing Medical University, Chongqing Key Laboratory of Pediatrics, National Clinical Research Center for Child Health and Disorders, Ministry of Education Key Laboratory of Child Development and Disorders, Chongqing, 400010 China

**Keywords:** HCC, RPL27A, Prognosis, Co-expression genes, Immune infiltration

## Abstract

**Background:**

Hepatocellular carcinoma (HCC) is one of the most common cancers in the digestive system with rapid progression and poor prognosis. Recent studies have shown that RPL27A could be used as a biomarker for a variety of cancers, but its role in HCC is not clear.

**Method:**

We analyzed the expression of RPL27A in the pan-cancer analysis and analyzed the relationship between the expression of RPL27A and the clinical features and prognosis of patients with HCC. We evaluated the expression difference of RPL27A in HCC tissues and paired normal adjacent tissues using immunohistochemistry. Furthermore, we analyzed the co-expression genes of RPL27A and used them to explore the possible mechanism of RPL27A and screen hub genes effecting HCC. In addition, we studied the role of RPL27A in immune infiltration and mutation.

**Results:**

We found that the expression level of RPL27A increased in a variety of cancers, including HCC. In HCC patients, the high expression of RPL27A was related to progression and poor prognosis as an independent predictor. We also constructed a protein interaction network through co-expression gene analysis of RPL27A and screened 9 hub genes. Enrichment analysis showed that co-expression genes were associated with ribosome pathway, viral replication, nuclear-transcribed mRNA catabolic process, and nonsense-mediated decay. We found that the expression level of RPL27A was closely related to TP53 mutation and immune infiltration in HCC.

**Conclusion:**

RPL27A might become a biomarker in the diagnosis, treatment, and follow-up of patients with HCC.

## Introduction

Liver cancer which mainly includes hepatocellular carcinoma (HCC) (75–85%) and intrahepatic cholangiocarcinoma (10–15%) ranks seventh in the incidence and fifth in mortality of cancers in the world [1]. Although many prevention measures such as hepatitis B virus (HBV) vaccination have been taken and treatment methods have been continuously enriched, the increasing trend of morbidity and mortality of HCC has not been effectively curbed. HCC, as one of the three most common and highest mortality cancers in the digestive system, has been a focus of public health worldwide for a long time [2]. Early detection, early diagnosis, and early treatment are very important for the management of HCC, and effective biomarkers may be one of the breakthroughs.

Cancer cells tend to have strong proliferative ability and high metabolic levels. Protein, as the executor of biological function, is related to the occurrence and progression of cancer. The ribosome which is composed of ribosome RNAs (rRNA) and ribosomal proteins (RPs) plays an important role in intracellular protein biosynthesis, and RPs genes are the most highly expressed genes in most cell types, especially in cancer cells [3–5]. The abnormal expression of RPs in some cancers can promote tumor progression such as proliferation and metastasis and could act as a tumor biomarker [6–9]. RPL27A, as one kind of large subunit RPs, belongs to the universal ribosomal protein uL15 family and is closely correlated with some cancers, such as breast cancer and colorectal cancer [10, 11]. Weighted gene co-expression network analysis and multi-dataset verification showed RPL27A was highly expressed in HCC [12], but there were no further studies to explore the value of RPL27A in HCC.

In this study, we took full advantage of The Cancer Genome Atlas (TCGA) database and tissue microarray (TMA) to analyze the role of RPL27A in evaluating the prognosis of patients with HCC. We performed co-expression gene analyses, function enrichment analyses, and immune cell infiltration analyses to explore the role of RPL27A in HCC.

## Methods

### Pan-cancer analysis of RPL27 and expression of RPL27A in HCC

*TIMER* (https://cistrome.shinyapps.io/timer/) is a web server for comprehensive analysis of tumor-infiltrating immune cells based on the TCGA database [13, 14]. After submitting the gene of interest in the *DiffExp* module of *TIMER*, the expression levels of the gene between tumor and adjacent normal tissues in many kinds of cancers can be obtained.

*UALCAN* (http://ualcan.path.uab.edu/) is an interactive web resource and allows users to verify the value of genes of interest in the target cancer based on the TCGA database [15]. We analyzed the relationship of RPL27A and clinicopathologic characteristics such as grades and stages in HCC.

### Survival analysis about RPL27A in HCC

Survival curves were plotted in *Kaplan–Meier Plotter* (https://kmplot.com/analysis/) which is an online tool to study the effect of the gene of interest on survival in the target cancer based on the TCGA database [16]. We used survival curves to evaluate the role of RPL27A in the prognosis of HCC.

### RPL27A expression levels in HCC

We used TMA to further verify the expression level and prognostic role of RPL27A in patients with HCC. TMA containing 180 tissues, including 92 HCC tissues and 88 peritumoral normal liver tissues, was purchased from *Outdo Biotech* (Shanghai, China). The above tissues were from 92 patients with HCC, of which 88 pairs were paired samples. TMA was processed for immunohistochemistry (IHC) according to standard procedures. Primary antibody against RPL27A was purchased from *Huabio* (ER64831, 1:1300, Hangzhou, China). We used the *Fromowitz* semiquantitative method to assess the expression level of RPL27A in IHC [17]. In short, the TMA was used to analyze the difference in RPL27A expression between HCC tissues and peritumoral liver tissues and evaluate the relationship between RPL27A and prognosis of HCC patients.

### Co-expression genes analysis of RPL27A

*LinkedOmics* (http://www.linkedomics.org/) includes multi-omics data from TCGA and Clinical Proteomics Tumor Analysis Consortium (CPTAC) [18]. We performed co-expression gene analysis of RPL27A and plotted heat maps and correlation scatter plots by *LinkedOmics*. We screened out the genes strongly correlated with RPL27A, which were used for Gene Ontology (GO), Kyoto Encyclopedia of Genes and Genomes (KEGG) enrichment analyses, and gene set enrichment analyses (GSEA). In addition, the above genes were used to construct the protein–protein interaction (PPI) network using the *STRING* (https://string-db.org/), whose analysis result was imported into *Cytoscape* (https://cytoscape.org/) to screen hub genes. In brief, we used the above methods to explore the possible mechanism of RPL27A in HCC.

### The role of RPL27A in immune infiltration

We explored the correlation between the expression of RPL27A and abundance of immune infiltrates (B cells, CD4 + T cells, CD8 + T cells, neutrophils, macrophages, and dendritic cells) in pan-caner analysis including HCC through the *Gene* module in *TIMER*.

### Mutation analysis about RPL27A

The mutation type of RPL27A was analyzed using the *cBioPortal* database (https://www.cbioportal.org/) which is a visual tool for studying and analyzing cancer gene data and can help researchers understand epigenetics, gene mutation, and proteomics about cancer histology and cytology studies [19, 20]. In addition, we analyzed the relationship between the expression of RPL27A and mutation of tumor protein 53 (TP53) using *UALCAN*.

### Statistical analysis

The expression data of RPL27A in *UALCAN* and *TIMER* is the continuous data with the Gauss distribution, and *T*-test was used to analyze the differences of RPL27A expression between tumor and peritumoral groups and between different clinicopathologic characteristic groups. For TMA, *chi-square* test was used to analyze the differences in baseline features among groups with different RPL27A expression levels, and *McNemar’s chi-squared* test was used to access the difference in RPL27A expression between paired samples. *Kaplan–Meier* method was used to plot survival curves and *log-rank* method was used to analyze prognosis differences among groups with different expression levels of RPL27A. To explore the prognostic value of RPL27A in HCC, the factors with *P* value < 0.05 in the univariate *Cox* regression analysis were used in the multivariate *Cox* regression analysis, and those with *P* value < 0.05 in the multivariate analysis were considered as prognostic factors. We used the area under the curve (AUC) by plotting receiver operator characteristic (ROC) curves to further assess the prognostic value of RPL27A in HCC. *Spearman* correlation analysis was used in co-expression gene analysis of RPL27A in *LinkedOmics.* Immune infiltration analysis of RPL27A in HCC was estimated by TIMER algorithm in *TIMER*. *P* value < 0.05 was considered statistically significant and *Spearman* correlation coefficient ≥ 0.6 was considered a strong correlation. GO, KEGG, GSEA, and ROC curves plotting were performed by *R* software (https://www.r-project.org/) using *survival*, *survminer*, *timeROC*, *rms*, *tableone*, *clusterProfiler*, and *ggplot2* packages.

## Results

### The relationship between RPL27A expression and progression in HCC

**Fig. 1:**
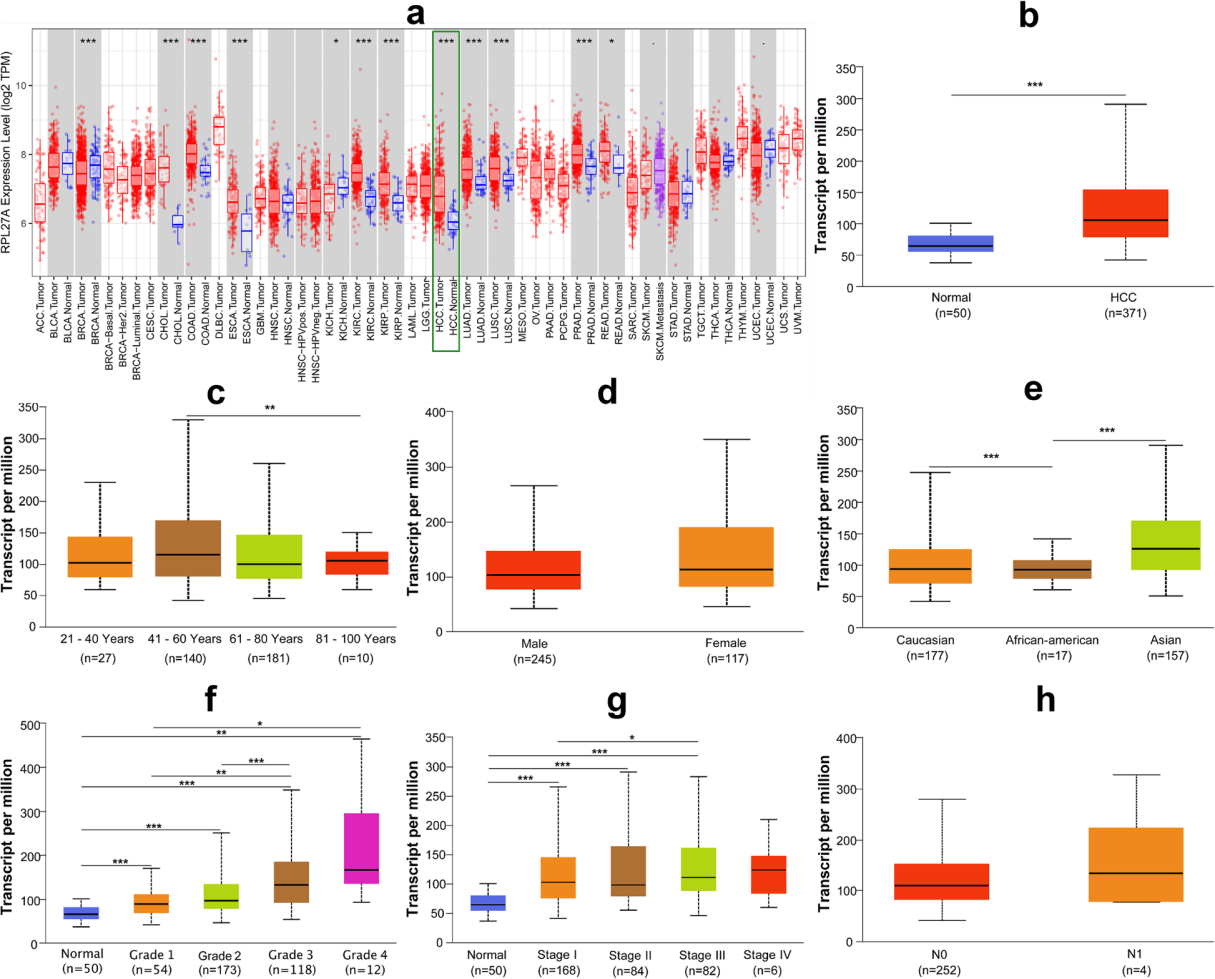
The expression of RPL27A in pan-cancer and HCC based on the TCGA database. **a** The pan-cancer analysis of RPL27A. **b** The expression of RPL27A between HCC and normal tissues. **c**–**h** The expression of RPL27A in HCC among different age, gender, race, grade, stage, and nodal metastasis status groups. *P* value significant codes: 0 ≤ *** < 0.001 ≤ ** < 0.01 ≤ * < 0.05

**Fig. 2:**
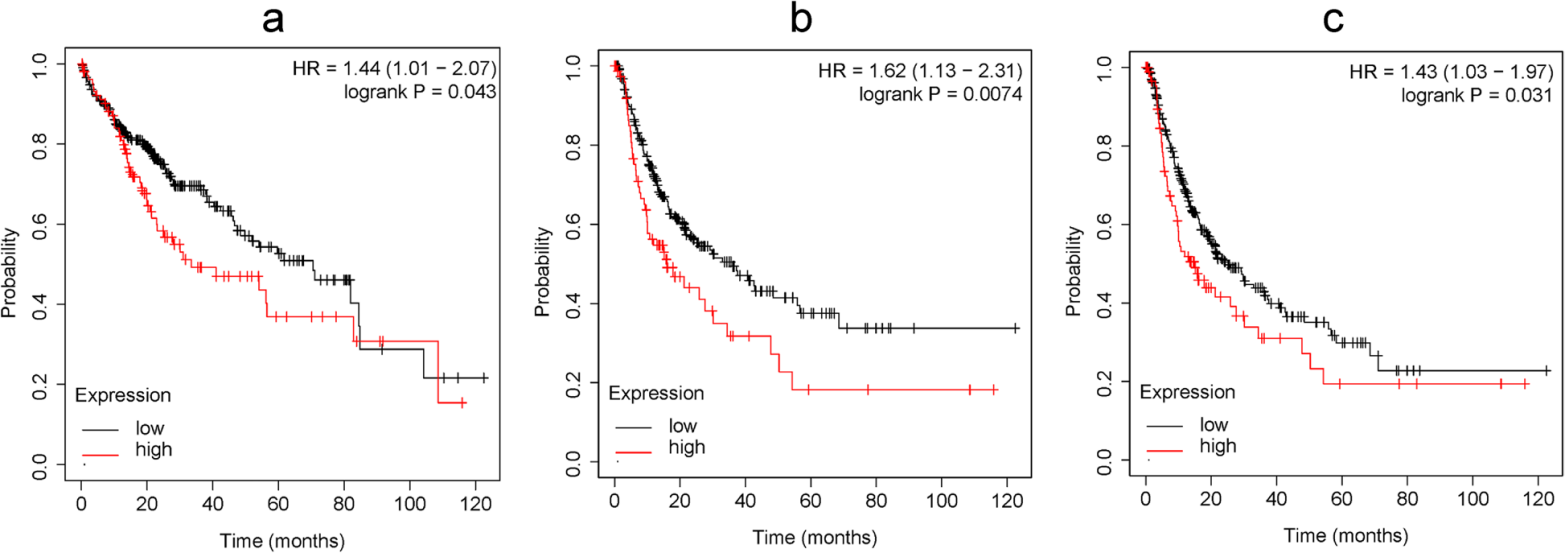
Survival curves among different levels of RPL27A expression in HCC based on the TCGA database. **a** OS of HCC patients among groups with different expression levels of RPL27A. **b** PFS of HCC patients among groups with different expression levels of RPL27A. **c** DFS of HCC patients among groups with different expression levels of RPL27A

We found that RPL27A was significantly highly expressed in many kinds of cancers (*P* < 0.05), including breast invasive carcinoma (BRCA), cholangiocarcinoma (CHOL), colon adenocarcinoma (COAD), esophageal carcinoma (ESCA), kidney chromophobe (KICH), kidney renal clear cell carcinoma (KIRC), kidney renal papillary cell carcinoma (KIRP), HCC, lung adenocarcinoma (LUAD), lung squamous cell carcinoma (LUSC), prostate adenocarcinoma (PRAD), and rectum adenocarcinoma (READ) (Fig. 1a). The expression of RPL27A in HCC tissues was significantly higher than that in normal tissues (*P* < 0.05) (Fig. 1a, b). In HCC patients, the expression of RPL27 was significantly increased in 41–61 years old, Asian, Caucasian, greater grade, and American Joint Committee on Cancer (AJCC) stage III groups (*P* < 0.05) (Fig. 1c, e–g). However, its expression levels were not significantly different between different gender groups and nodal metastasis status groups in HCC patients (*P* > 0.05) (Fig. 1d, h). Survival curves of overall survival (OS), progression-free survival (PFS) rate, and disease-free survival (DFS) showed that HCC patients with higher level of RPL27A expression had poorer survival outcome (*P* < 0.05) (Fig. 2a–c).

**Fig. 3:**
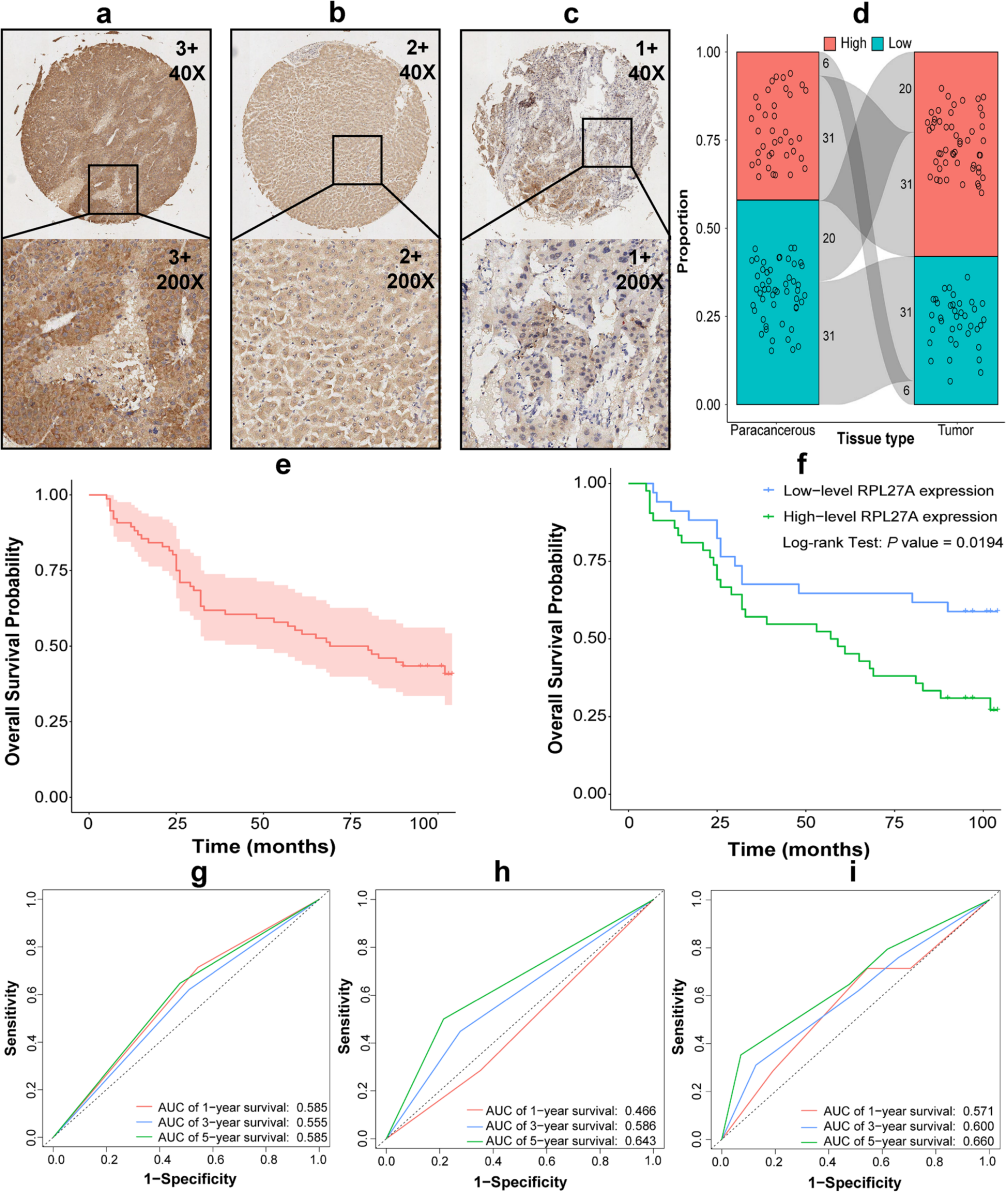
RPL27A expression in HCC in TMA. **a**–**c** Examples of different RPL27A IHC scores in TMA. **d** The difference of RPL27A expression among HCC and paracancerous tissues using *McNemar’s chi-squared* test. **e** Survival curve of HCC patients. **f** Survival curves of HCC patients among groups with different expression levels of RPL27A. **g**–**i** ROC curves drawn using RPL27A only, AJCC stage only, and both RPL27A and AJCC stage for evaluating the prognosis of patients with HCC

**Fig. 4:**
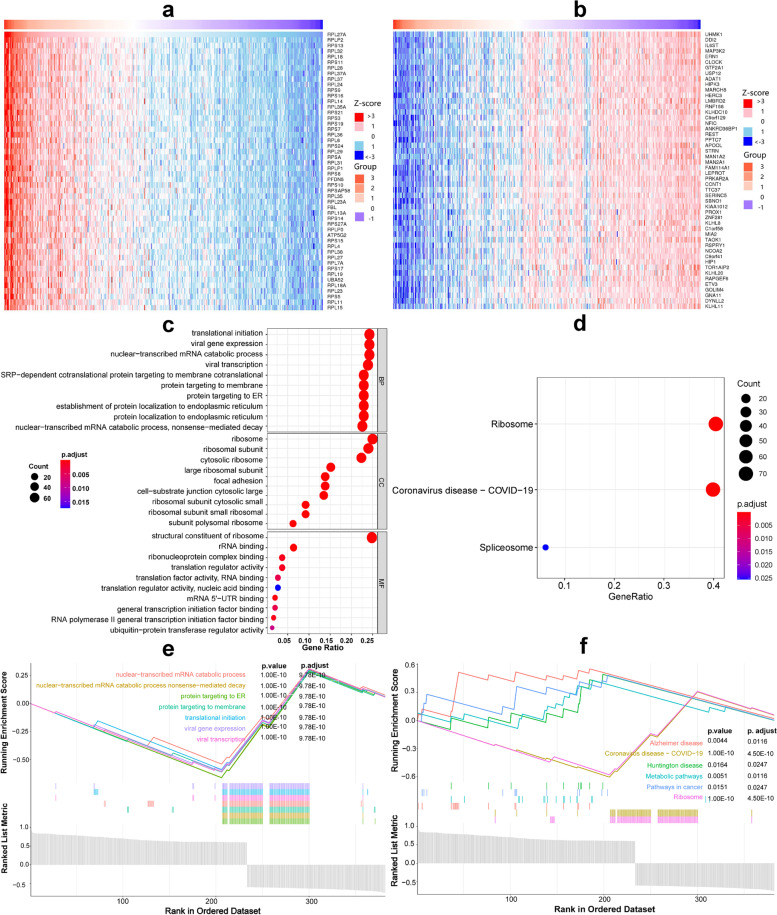
Co-expression gene analysis of RPL27A in HCC based on TCGA. **a**, **b** The heat map of the top 50 significant genes of positively correlated with and negatively correlated with RPL27A in HCC. **c** The GO enrichment analysis of significant genes of strongly correlated with RPL27A in HCC. **d** The KEGG enrichment analysis of significant genes of strongly correlated with RPL27A in HCC. **e**, **f** The GSEA BP and pathway enrichment analysis of significant genes of strongly correlated with RPL27A in HCC

We further verified the RPL27A expression level in HCC using IHC in TMA. Excluding patients with insufficient clinical data, we analyzed the clinicopathological data of 76 patients with HCC. We found that there were different expression levels of RPL27A in all tumor and peritumoral tissues in TMA. So, 1 + and 2 + were regarded as low expression levels, while 3 + was regarded as high expression levels in this study (Fig. 3a). We found that there was no significant difference in baseline characteristics including age, gender, hepatitis B surface antigen (HBsAg), AFP, tumor size, cirrhosis, grade and AJCC stage, and programmed cell death protein 1 (PD-1) among groups with different RPL27 expression levels (*P* > 0.05) (Table 1). We found that RPL27A mainly expresses in the cytoplasm (Fig. 3a–c), and the expression of RPL27A in HCC tissues was significantly higher than that in paratumoral tissues (*P* = 0.011) (Fig. 3d, the gray shadow connects the paired samples). The results of univariate and multivariate *Cox* regression analyses showed that high expression level of RPL27A (HR = 2.123, 95%CI 1.115–4.046, *P* = 0.043) and stage II and III (HR = 1.890, 95%CI 1.020–3.503, *P* = 0.022) were independent predictors of shorter OS (Table 2, Fig. 4e, f). We further found that the stability of RPL27A was better than the AJCC stage in evaluating the prognosis of patients with HCC (Fig. 3g vs Fig. 3h), and RPL27A could be a good addition for the AJCC stage (Fig. 3i).

**Table 1:** Clinicopathological characteristics of patients with HCC between groups with different RPL27A expression levels

	**Overall (*n* = 76)**	**Low-level (*n* = 34)**	**High-level (*n* = 42)**	***P* value**
**Age of diagnosis**				
≤ 50 years	32 (42.1%)	16 (47.1%)	16 (38.1%)	0.580
> 50 years	44 (57.9%)	18 (52.9%)	26 (61.9%)	
**Gender**				
Male	70 (92.1%)	30 (88.2%)	40 (95.2%)	0.485
Female	6 (7.9%)	4 (11.8%)	2 (4.8%)	
**HBsAg**				
Negative	15 (19.7%)	7 (20.6%)	8 (19.0%)	1.000
Positive	61 (80.3%)	27 (79.4%)	34 (81.0%)	
**AFP**				
Negative	32 (42.1%)	18 (52.9%)	14 (33.3%)	0.137
Positive	44 (57.9%)	16 (47.1%)	28 (66.7%)	
**Tumor size**				
≤ 50 mm	53 (69.7%)	23 (67.6%)	30 (71.4%)	0.916
> 50 mm	23 (30.3%)	11 (32.4%)	12 (28.6%)	
**Cirrhosis**				
Negative	9 (11.8%)	6 (17.6%)	3 (7.1%)	0.293
Positive	67 (88.2%)	28 (82.4%)	39 (92.9%)	
**Grade**				
Grades I and III	34 (44.7%)	18 (52.9%)	16 (38.1%)	0.288
Grade II	42 (55.3%)	16 (47.1%)	26 (61.9%)	
**Stage**				
I	50 (65.8%)	23 (67.6%)	27 (64.3%)	0.949
II and III	26 (34.2%)	11 (32.4%)	15 (35.7%)	
**PD-1**				
Negative	38 (50.0%)	18 (52.9%)	20 (47.6%)	0.818
Positive	38 (50.0%)	16 (47.1%)	22 (52.4%)

**Table 2:** Univariate and multivariate *Cox* regression analysis in patients with HCC

	**Univariate analysis**		**Multivariate analysis**	
	**HR (95% CI)**	***P* value**	**HR (95% CI)**	***P* value**
**Age at diagnosis**				
≤ 50 years	1.000 [Reference]	-	-	-
> 50 years	1.358 [0.734, 2.512]	0.329	-	-
**Gender**				
Male	1.000 [Reference]	-	-	-
Female	0.442 [0.107, 1.829]	0.260	-	-
**HBsAg**				
Negative	1.000 [Reference]	-		
Positive	1.097 [0.509, 2.365]	0.814		
**AFP**				
Negative	1.000 [Reference]	-	-	-
Positive	1.512 [0.817, 2.797]	0.188	-	-
**Tumor size**				
< 50 mm	1.000 [Reference]	-	-	-
≥ 50 mm	1.676 [0.904, 3.108]	0.101	-	-
**Cirrhosis**				
Negative	1.000 [Reference]	-	-	-
Positive	1.589 [0.568, 4.446]	0.377	-	-
**Grade**				
Grade I and II	1.000 [Reference]	-	1.000 [Reference]	-
Grade III	1.970 [1.063, 3.650]	0.031	1.733 [0.930, 3.228]	0.083
**AJCC stage**				
I	1.000 [Reference]	-	1.000 [Reference]	-
II and III	1.861 [1.018, 3.402]	0.044	1.890 [1.020, 3.503]	0.022
**RPL27A**				
Low-level	1.000 [Reference]	-	1.000 [Reference]	-
High-level	2.137 [1.131, 4.037]	0.019	2.123 [1.115, 4.046]	0.043
**PD-1**				
Negative	1.000 [Reference]	-	-	-
Positive	0.689 [0.379, 1.253]	0.222	-	-

### The co-expression genes analysis of RPL27A in HCC

In the co-expression gene analysis, 367 strong related genes were screened out, of which the top 50 positively and negatively correlated genes were showed in the heatmaps respectively (Fig. 4a, b). The above 367 genes were used to perform GO and KEGG enrichment analyses (Fig. 4c, d). We found that these genes were mainly enriched in biological process (BP) such as translational initiation, viral gene expression, nuclear-transcribed mRNA catabolic process, and nonsense-mediated decay (NMD); in cellular component (CC) such as ribosome, ribosomal subunit, and cytosolic ribosome; in molecular function (MF) such as structural constituent of ribosome; and in pathway such as ribosome, COVID-19, and spliceosome. We found that these genes were negatively associated with 7 BPs mainly nuclear-transcribed mRNA catabolic process, protein targeting, viral gene expression, and transcription through GSEA, because the curve was funnel-shaped (Fig. 4e). These genes were positively associated with pathways in cancer through GSEA (Fig. 4f). Therefore, RPL27A may play a potential role in HCC through regulating the above functions or pathways.

**Fig. 5:**
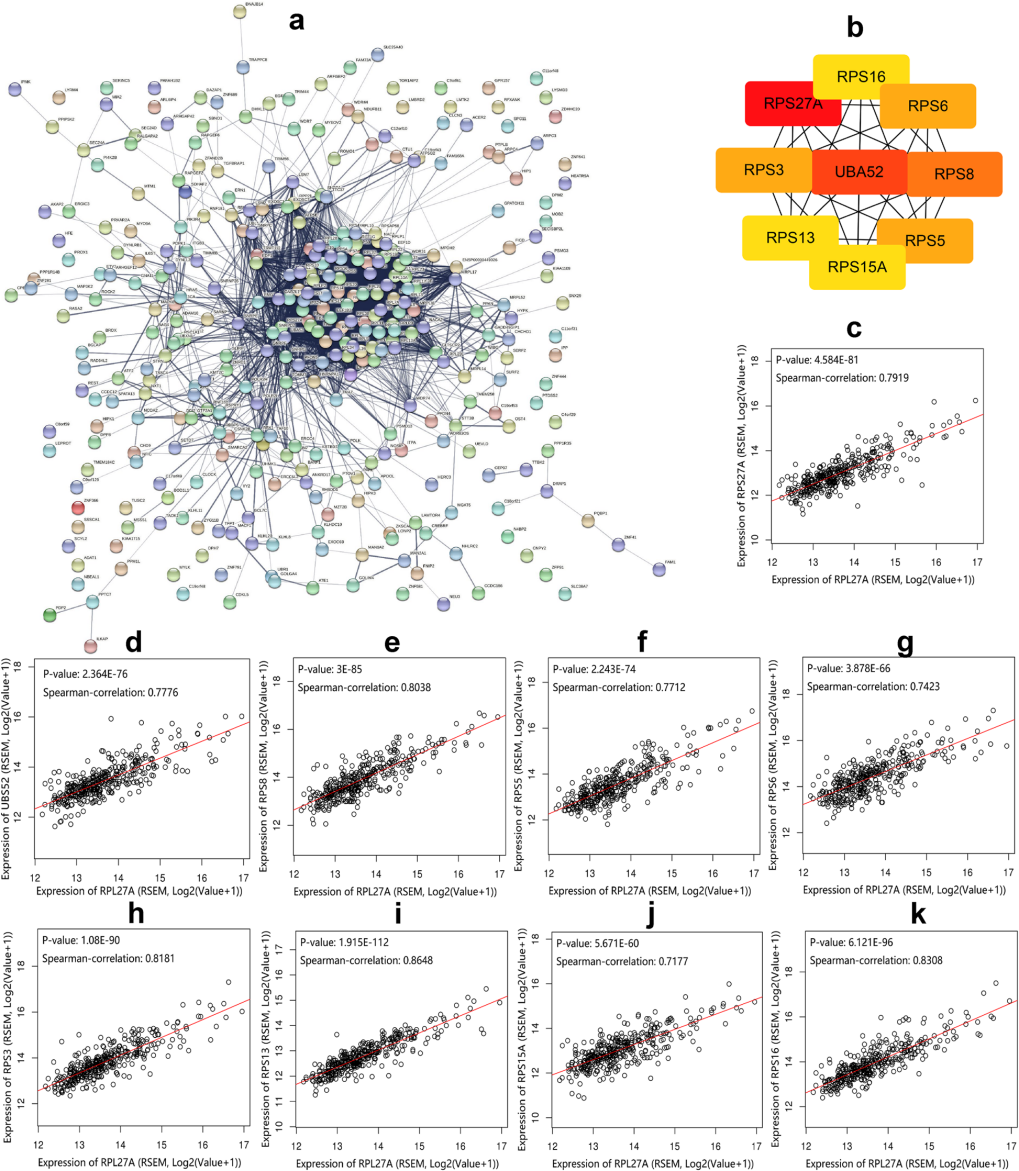
PPI analysis of co-expression genes related with RPL27A in HCC. **a** The PPI network of significant genes of strongly correlated with RPL27A in HCC. **b** Network of top 9 hub gene strongly correlated with RPL27A in HCC. **c–k** Scatter plots of top 9 hub genes and RPL27A based on TCGA

The above 367 genes were used to construct the PPI network in *STRING*, in which there were 351 nodes and 4689 edges under minimum required interaction score of 0.400 (Fig. 5a). The analysis result was imported into *Cytoscape* to be calculated in the *cytoHubba* app. There were 9 hub genes screened in the method of degree, including RPS27A, UBA52, RPS8, RPS5, RPS6, RPS3, RPS13, RPS15A, and RPS16 (Fig. 5b, Table 3). We plotted scatter maps of RPL27A and 9 hub genes, which showed that the expression of RPL27A was positively correlated with those of above hub genes (Fig. 5c–k). In short, the above hub genes may be closely related to the mechanism of RPL27A in HCC.

**Table 3:** Top 9 hub genes correlated with RPL27A in HCC based on the TCGA database

Gene	Spearman	*P* value	FDR (BH)	Degree
RPS27A	0.7919	4.58E − 81	2.61E − 78	113
UBA52	0.7776	2.36E − 76	1.05E − 73	105
RPS8	0.8038	3.00E − 85	2.30E − 82	100
RPS5	0.7712	2.24E − 74	9.31E − 72	99
RPS6	0.7423	3.88E − 66	1.15E − 63	99
RPS3	0.8181	1.08E − 90	1.34E − 87	99
RPS13	0.8648	1.91E − 112	1.27E − 108	98
RPS15A	0.7177	5.67E − 60	1.31E − 57	98
RPS16	0.8308	6.12E − 96	1.02E − 92	98

### The relationship between RPL27A and immune infiltration in HCC

**Fig. 6:**
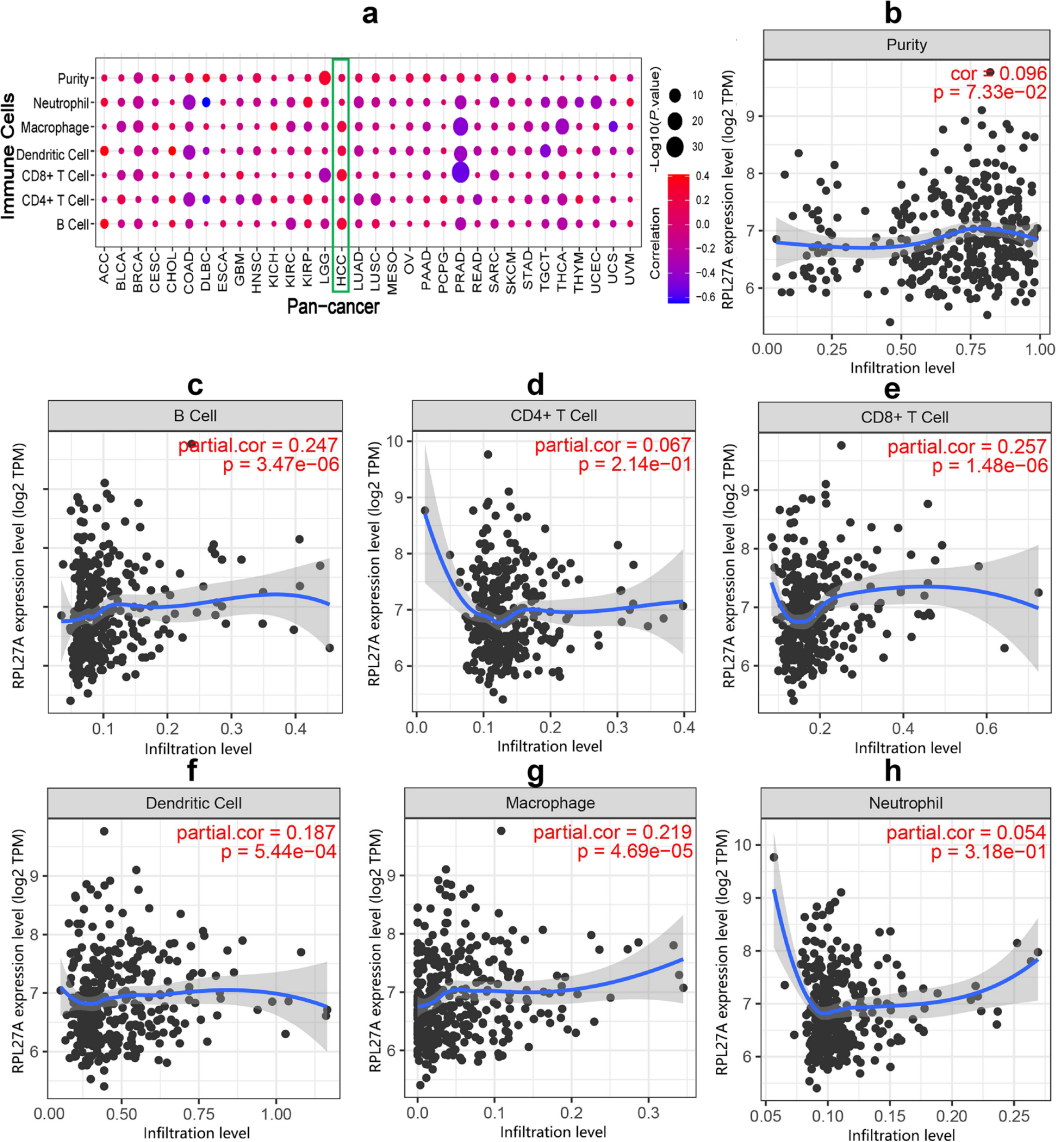
Immune infiltration analysis of RPL27A in HCC based on TCGA. **a** Immune infiltration associated with RPL27A in the pan-cancer analysis. **b**–**h** Immune infiltration associated with RPL27A in HCC

In the pan-cancer analysis based on the TCGA database, we used *TIMER* to study the role of RPL27A in immune infiltration. We found that expression of RPL27A was significantly correlated with immune infiltration in many kinds of cancers such as PRAD, thymoma (THCA), COAD, uterine corpus endometrial carcinoma (UCEC), BRCA, KIRC, LUSC, LUAD, HCC, head-neck squamous cell carcinoma (HNSC), and bladder urothelial carcinoma (BLCA) (Fig. 6a). In addition, we evaluated the relationship between RPL27A and immune infiltration in HCC, including B cells, CD4 + T cells, CD8 + T cells, neutrophils, macrophages, and dendritic cells (Fig. 6b–h). Among them, RPL27A was significantly positively correlated with CD8 + T cells (partial.cor = 0.257, *P* = 1.48E − 06), B cells (partial.cor = 0.247, *P* = 3.37E − 06), macrophages (partial.cor = 0.219, *P* = 4.69E − 05), and dendritic cells (partial.cor = 0.187, *P* = 5.44E − 04).

### RPL27A and mutation in HCC

**Fig. 7:**
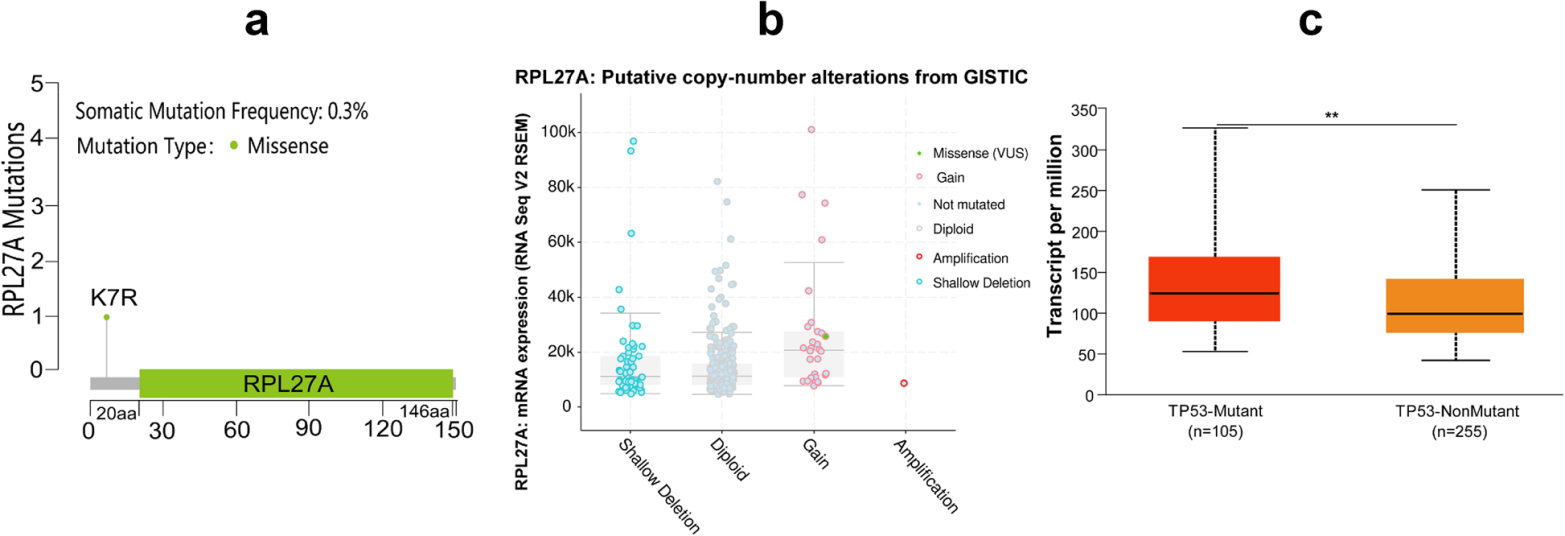
Mutation analysis of RPL27A in HCC based on TCGA. **a**, **b** Mutation associated with RPL27A in HCC. **c** The expression of RPL27A in HCC among different TP53 mutation status groups. *P* value significant codes: 0 ≤ *** < 0.001 ≤ ** < 0.01 ≤ * < 0.05

We performed mutation analysis about RPL27A using the *cBioPortal* database. The frequency of somatic mutations was 0.3% (1/360) in HCC patients from the TCGA database, the mutation type was missense, and copy number alteration was gained (Fig. 7a, b). We found that the expression level of RPL27A in the TP53 mutation group was significantly higher than that in the TP53 non-mutation group in HCC (*P* < 0.05) (Fig. 7c).

## Discussion

With the application of public health measures such as HBV immunization, treatment of chronic HBV and HCV, and reduction of aflatoxin exposure, and the continuous development of treatment technology for HCC, human beings seem to be increasingly optimistic in the face of HCC [2, 21–23]. However, the global burden caused by HCC is still an arduous challenge, attributed to the large patient number, alcoholism, obesity, diabetes, nonalcoholic fatty liver disease, and other non-viral factors [2, 23]. HCC patients are often at an advanced stage when they are diagnosed, and they have a poor prognosis in the world with a very poor 5-year survival rate of only 18% and a 5-year recurrence rate of more than 60% [1, 2, 23–25]. Effective biomarkers, targeted therapy, and immunotherapy may be breakthroughs in the treatment of HCC, which have become hot research fields.

We found that the expression of RPL27A was significantly increased in a variety of cancers, suggesting that RPL27A may play an important role in the occurrence and development of cancers. Many studies have confirmed that RPL27A could be a potential biomarker of lung cancer, triple-negative breast cancer, squamous cervical cancer, colorectal cancer, and KIRC [8, 10, 11, 26–28]. We found that the expression level of RPL27A in HCC tissues was significantly higher than that in normal tissues. Our results showed that the high expression of RPL27A was related to the late stage and high grade of HCC, so RPL27A may be considered as a complementary biomarker of the occurrence and development for patients with HCC. Furthermore, we found that the prognosis of the high RPL27A expression group was worse than that of the low RPL27A expression group, and RPL27A could perform better than the AJCC stage in evaluating the prognosis of patients with HCC. Therefore, RPL27A could play a good auxiliary role and addition in the follow-up of patients with HCC.

To explore the possible mechanism of RPL27A in HCC, we analyzed the co-expression genes of RPL27A and the relationship between RPL27A and TP53 mutation. We found these genes mainly enriched the ribosome pathway and were mainly involved in viral gene expression, viral transcription, NMD, and proteins localization to endoplasmic reticulum (ER). NMD is a very conservative mRNA surveillance pathway to ensure the stability and quality of transcripts [29]. However, some cancers can exploit NMD to inactivate tumor suppressor genes, and NMD is involved in tumor adaptation to the harsh tumor microenvironment (TME), such as various stresses including hypoxia and reactive oxygen species [30–32]. The imbalance of RPs in cancer may be involved in the pathogenesis of ER stress [33]. In addition, active transcription and translation of HBV favor its replication and are crucial in its pathogenic and carcinogenic mechanism [34]. In this study, all hub genes from the co-expression gene analysis of RPL27A in HCC belong to RPs. Many studies also supported the role of our hub genes in the occurrence and development of HCC [35–40]. Furthermore, we found the HCC patients with TP53 mutation had significantly higher expression level of RPL27A and co-expression genes of RPL27A could regulate the activity of the ubiquitin-protein transferase. TP53 as one of the most important tumor suppressor genes is involved in cell differentiation, cell cycle regulation, and apoptosis, and TP53 loss-of-function is associated with cancer progression and poor prognosis in HCC patients [41]. Mutation, NMD, and ubiquitin can cause the loss of tumor suppressor function of TP53 [42–44]. In short, RPL27A might affect HCC by the above direct or indirect pathways which could be directions of HCC research in the future.

Immune microenvironment is one important part of TME, and immune escape mechanism is one of the most important hallmarks of cancer [45]. In recent years, immunotherapy has shown satisfactory results in some cancers and has become a promising method for cancer treatment, including immune checkpoint modulators and adoptive immune cells [46]. We found that RPL27A expression was related to a variety of immune cells in HCC, including B cells, CD4 + T cells, CD8 + T cells, neutrophils, macrophages, and dendritic cells. Studies have shown that tumor-infiltrating immune cells can behave as either tumor-promoting or tumor-suppressive, possibly associated with the dysfunction of immune cells caused by themselves or tumors [46, 47]. Regulatory B cells, a subset of B cells, are associated with advanced stage and poor prognosis and can mediate immune escape of HCC [48, 49]. Many studies have found that CD4 + T cells are closely related to the occurrence and development of HCC, and the injury or depletion of CD4 + T cells can promote the above processes [50, 51]. Like CD4 + T cells, CD8 + T cell dysfunction can promote the growth and metastasis of HCC and is closely related to the prognosis of patients with HCC [52, 53]. In addition, tumor-associated macrophages are associated with drug resistance, cancer progression, and poor prognosis, including HCC [54–56]. Altogether, RPL27A might affect HCC by immune infiltration, but further research are needed to confirm it.

Currently, this study and our understanding of RPL27A in HCC have several limitations. First, this study mainly used several online databases to explore the possible mechanism of the effect of RPL27A on HCC, but lack of further studies in vitro or in vivo to verify our hypotheses. Second, although this study through several online databases and TMA showed that RPL27A may be a prognostic marker for patients with HCC, more basic research and large sample clinical studies should be performed to further confirm if it was to be used for patients. In the future, we will extend the present studies to explore the role of RPL27A in HCC.

## Conclusion

In this study, we discussed the role of RPL27A in HCC from multiple angles and speculated that it may become a biomarker in the diagnosis, treatment, and follow-up of patients with HCC, but further studies are required to verify it.

## Data Availability

The authors ensure that the data analyzed in the research is publicly available. The data can be found as follows: *TIMER* (https://cistrome.shinyapps.io/timer/), *UALCAN* (http://ualcan.path.uab.edu/), *Kaplan–Meier Plotter* (https://kmplot.com/analysis/), *LinkedOmics* (http://www.linkedomics.org/), *STRING* (https://string-db.org/), *Cytoscape* (https://cytoscape.org/), *cBioPortal* database (https://www.cbioportal.org/).

## Abbreviations

AJCC: American Joint Committee on Cancer
AUC: Area under the curve
BLCA: Bladder urothelial carcinoma
BP: Biological process
BRCA: Breast invasive carcinoma
CC: Cellular component
CHOL: Cholangiocarcinoma
COAD: Colon adenocarcinoma
CPTAC: Clinical Proteomics Tumor Analysis Consortium
DFS: Disease-free survival
ER: Endoplasmic reticulum
ESCA: Esophageal carcinoma
GO: Gene Ontology
GSEA: Gene set enrichment analysis
HBV: Hepatitis B virus
HCC: Hepatocellular carcinoma
HNSC: Head-neck squamous cell carcinoma
IHC: Immunohistochemistry
KEGG: Kyoto Encyclopedia of Genes and Genomes
KICH: Kidney chromophobe
KIRC: Kidney renal clear cell carcinoma
KIRP: Kidney renal papillary cell carcinoma
LUAD: Lung adenocarcinoma
LUSC: Lung squamous cell carcinoma
MF: Molecular function
NMD: Nonsense-mediated decay
OS: Overall survival
PD-1: Programmed cell death protein 1
PFS: Progression-free survival
PRAD: Prostate adenocarcinoma
READ: Rectum adenocarcinoma
ROC: Receiver operator characteristic
RPs: Ribosomal proteins
rRNA: Ribosome RNAs
TCGA: The Cancer Genome Atlas
THCA: Thymoma
TMA: Tissue microarray
TME: Tumor microenvironment
TP53: Tumor protein 53
UCEC: Uterine corpus endometrial carcinoma
